# Articular cartilage mineralization in osteoarthritis of the hip

**DOI:** 10.1186/1471-2474-10-166

**Published:** 2009-12-29

**Authors:** Martin Fuerst, Oliver Niggemeyer, Lydia Lammers, Fritz Schäfer, Christoph Lohmann, Wolfgang Rüther

**Affiliations:** 1 Department of Orthopedic Surgery, University Medical Center Hamburg-Eppendorf, Clinic Bad Bramstedt, Martinistr 52, 20465 Hamburg, Germany; 2 Institute of Mineralogy, University Hospital Münster, Waldeyerstr 30, 48149 Münster, Germany; 3 Breast Center, University Hospital Kiel, Arnold-Heller-Str 3, 24108 Kiel, Germany

## Abstract

**Background:**

The aim of this study was to examine the frequency of articular cartilage calcification in patients with end-stage hip OA. Further, its impact on the clinical situation and the OA severity are analyzed.

**Methods:**

Eighty patients with OA of the hip who consecutively underwent total hip replacement were prospectively evaluated, and 10 controls were included. The patients' X-rays were analyzed for the presence of articular cartilage mineralization. A Harris Hip Score (HHS) was preoperatively calculated for every patient.

Slab specimens from the femoral head of bone and cartilage and an additional square centimeter of articular cartilage from the main chondral defect were obtained from each patient for analysis of mineralization by digital contact radiography (DCR). Histological grading was also performed. In a subset of 20 patients, minerals were characterized with an electron microscope (FE-SEM).

**Results:**

Calcifications were seen in all OA cartilage and slab specimens using DCR, while preoperative X-rays revealed calcification in only 17.5%. None of the control cartilage specimens showed mineralization. There was a highly significant inverse correlation between articular cartilage calcification and preoperative HHS. Histological OA grade correlated positively with the amount of matrix calcification. FE-SEM analysis revealed basic calcium phosphate (BCP) as the predominant mineral; CPPD crystals were found in only two patients.

**Conclusions:**

Articular cartilage calcification is a common event in osteoarthritis of the hip. The amount of calcification correlates with clinical symptoms and histological OA grade.

## Background

OA is frequently associated with calcium crystal deposition. Two forms of calcium crystals have been studied in articular cartilage, meniscus tissue, and synovial fluid from patients with OA: calcium pyrophosphate dihydrate (CPPD) and basic calcium phosphate (BCP), including partly carbonate-substituted hydroxyapatite, tricalcium phosphate, and octacalcium phosphate [1-7].

Despite many reports showing the pathologic incidence of crystal deposition in osteoarthritic joints, there is much controversy about the frequency and significance of this association. In addition, most of the reports describe the frequency of calcinosis in the knee; only a few reports are about calcinosis of hip OA. Those findings indicate that calcinosis and OA are related, but the conclusion is less secure with the hip than with the knee [4,8].

A recent study by Mitsuyama et al. on 106 knees from 56 individual donors concluded that age rather than OA is the predominant factor driving progressive pathologic calcification in articular cartilage [9].

A clear understanding of the relationship between calcium crystal deposition and osteoarthritis is limited by the lack of a widely available and simple technique for detecting these crystals; scanning radiographs for chondrocalcinosis, the radiographic hallmark of calcium crystal deposition, is known to be insensitive [5]. Light and Polarizing light microscopy is the method of choice for detecting CPPD crystals, though only 20% of CPPD crystals are birefringent and some are too small to be detected by light or polarized light microscopy [1]. BCP crystals with a diameter of 0.1 nm to 1.0 nm are too small to be identified by light microscopy [2]. Diagnosis of mineralization with histological techniques is not specific and is often insensitive, especially in detecting single BCP crystals or small agglomerates of these minerals [2]. Alizarin red staining allows detection of calcium crystals and can be of interest in detecting BCP crystals when CPPD are not seen.

The clinical and pathological relevance of cartilage mineralization in patients with OA is not completely understood. In a recent study of 120 patients with OA of the knee, we could demonstrate that mineralization of articular cartilage by BCP is an indissociable process of OA and not only characterizes a specific subset of OA, but correlates significantly with clinical symptoms and the histological grade of the disease [10].

This study was performed on patients with end-stage OA undergoing total hip replacement, to test the hypothesis that calcification of the articular cartilage is a general phenomenon in patients with hip OA, that cartilage calcification has an impact on the clinical situation, and that calcification is associated with aggravated joint degeneration.

## Methods

This study was approved by the Ethics Committee of the Δrztekammer Schleswig-Holstein, Bad Segeberg, Germany. All patients included in this study gave full written informed consent for participation prior to the operative procedure.

Eighty patients undergoing total hip arthroplasty between October 2007 and February 2008 were included in this prospective study. All patients had primary osteoarthritis according to the ACR criteria for OA [11]. The indications for surgery were pain and limited function. The mean age of the patients was 69.9 years at the time of operation (± 0.86 SE, range 51-87); 53 were female and 27 were male (Table 1).

**Table 1:** Clinical data

OA patients	
Number of patients	80
Female/male	53/27
Mean age at time of operation	69.9 (± 0.86 years, range 51-87)
Left/right knee	36/44
Mean body mass index	28.6 (± 2.9, range 17.2-29.5)
Mean duration of the disease	3.2y (± 1.2 years, range 0.9-10.2)
Mean preoperative Harris Hip score	51.73 points (± 1.42, range 16-79)
Preoperative X-ray OA grade (Kellgren)	II: 4; III: 29; IV: 47

Clinical data on 80 patients with primary osteoarthritis of the hip are included in this study.

As a negative control, 10 patients with malignant bone tumors in the proximal femur (five osteosarcomas: 14, 15, 15, 16, and 18 years; two chondrosarcomas: 14 and 19 years; one metastasis of prostate cancer: 56 years; and two specimens from metastasis of lung cancer: 58 and 63 years) were included. During total or proximal femur replacement, cartilage was harvested from the unaffected hip joint following the same procedure used for the patients with OA.

Preoperatively, the OA patients were evaluated clinically and radiographically. The clinical and functional results were assessed with the Harris Hip Score (HSS) [12].

The patients' X-rays (standard AP and axial views) of the hip joint were analyzed for the presence of articular chondrocalcinosis. The X-rays of the affected hip were viewed by two orthopedic surgeons on the AP and axial views, building a consensus whether mineralization is visible or not. Periarticular mineralization was excluded.

Preoperative OA grades were evaluated according to the classification system of Kellgren and Lawrence [13].

During surgery, the entire resected femoral head was obtained from every patient. Two different techniques were used to obtain the cartilage specimens. First, the femoral head was cut into three 5 mm thick slab specimens of bone and cartilage in the coronal plane with the greatest possible diameter. These slab specimens included the mean weight-bearing area, the main chondral defect, and the surrounding cartilage of the defect. Additionally, about one square centimeter of articular cartilage from the main chondral defect was obtained. Only the upper half of the cartilage was cut off, to exclude subchondral bone artifacts. These two types of specimens were taken from every femoral head to extend the analyzed cartilage area and to minimize artifacts.

### Contact radiography (DCR)

First, each edge of the cartilage was trimmed and cleaned with a clean blade, removing bone debris from the sawing procedure to avoid contamination with bone dust from surgery or processing. The two types of cartilage specimens were used for detection and quantification of mineralization in every patient. All specimens were fixed in 10% neutral formalin prior to imaging. Three slab specimens and a tangential cut of cartilage from every patient were radiographed using a high-resolution digital mammography device (Hologic) operating at 25 KV in manual mode, usually at 3.8 mAs, and a film focus distance of 8 cm [10]. Mineralization could be viewed as regions within the cartilage matrix that appeared much brighter than the surrounding cartilage tissue. These digital images were analyzed by image-analysis software (ImageJ 1.32) [9,10]. This technique marks a given range of grey levels on the contact radiographs to identify areas for measurement. By adjusting the upper and lower bounds with a threshold utility, areas of calcification could be highlighted and used to calculate the percentage of mineralization in relation to the surrounding whole cartilage area. In some cases, there was a gradient in the cartilage due to varying thickness of the specimen. In these cases, the mineralized area was identified using a manual method, such as with a digital spray can tool. Whichever way the mineralized area was marked, the percentage of mineralization was determined by dividing the area of calcification by the whole area of cartilage. This gave a relative concentration of calcification for each specimen. The mean mineralization in the three slab specimens from each patient was calculated and used for further analysis.

### Histologic assessment

Histologic assessment was performed on the tangential cut cartilage specimen after the DCR procedure. The specimen was fixed in formalin, dehydrated by graded alcohols, and embedded in paraffin. Five-micrometer sections were cut, stained and evaluated using a modified Mankin grading system. The scores (on a scale of 0-14 points) for mild (1), moderate (2), and severe (3) OA changes in articular cartilage were 2-5, 6-9, and 10-14 points, respectively [14,15].

### Electron microscopy

Electron microscopy studies were performed on the tangential-cut specimens from a subset consisting of the first 20 patients without further selection. They were fixed in 4% formalin, dehydrated in ascending alcohol sequence, and transferred from 100% isopropanol to amylacetate followed by critical-point drying (CPD). Mineral cartilage characterization was achieved by means of a JEOL 6300F field emission electron microscope (FE-SEM) equipped with an energy-dispersive X-ray (EDX) system (Oxford Inca), operating in secondary electron (SE) and back-scattered electron (BSE) mode at various acceleration voltages from 0.5-20 KeV. With the EDX system, X-ray spectra from the material were collected and analyzed. The calcium phosphate ratio was determined by this method and distinguished CPPD (Ca/P-ratio: 1) from BCP crystals (for example, apatite, with a Ca/P-ratio of 1.67) [10].

### Statistical analysis

The correlation between two continuous parameters was analyzed by Spearman's correlation coefficient by rank test. Correlation between independent samples was determined by the Kruskal-Wallis test. The results were expressed as mean ± 2 standard error of mean (SE). A p value less than 0.05 was considered to be significant.

## Results

### DCR but not conventional X-ray reveals mineralization of the articular cartilage in all OA patients, with correlation to the clinical data

Systematic analysis of the preoperative X-rays of the hips revealed chondrocalcinosis in only 14 out of 80 patients (17.5%). However, when DCR was performed on both the tangential cut cartilage and the slab specimens, mineralization was found in all of them. The mean area of mineralization was 1.25% (± 0.14 SE, range 0.1-4.6%) for the tangential cut cartilage and 3.69% (± 0.31 SE, range 0.3-112.1) for the slab specimens.

**Figure 1:**
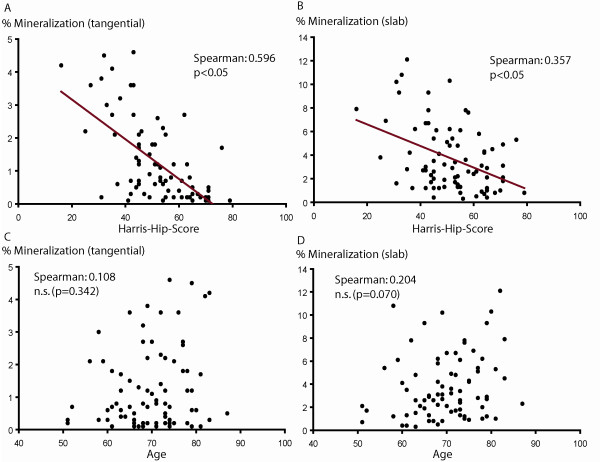
**Cartilage mineralization and clinical relationships**. Contact radiography (DCR) of tangential cut cartilage and slab specimens revealed mineralization in all articular cartilage specimens. The mean area of mineralization was 1.25% (± 0.14 SE, range 0.1-4.6%) for the tangential cut cartilage and 3.69% (± 0.31 SE, range 0.3-12.1) for the slab specimens. Scatter plots show a highly significant inverse correlation between HHS score and mean cartilage calcification for both the tangential cut articular cartilage and the slab specimens (A and B) (p < 0.01, p = 0.01). There was no correlation between patient age and the mean area of cartilage mineralization (C and D) (p = 0.342, p = 0.070).

As a part of the preoperative evaluation, clinical data were obtained from all 80 patients and an HHS score was calculated for each patient. The mean preoperative HHS was 51.73 points (± 1.42 SE, range 16-79). There was a significant inverse correlation between the preoperative HHS score and the mean area of cartilage mineralization for both the tangential cut articular cartilage and the slab specimens (p < 0.05). Scatter plots show a significant inverse relationship between HHS score and mean area of cartilage mineralization (Figures 1A and 1B). In other words, patients with a large amount of articular cartilage calcification showed worse preoperative clinical results compared with patients who presented with only a small amount of minerals in their articular cartilage.

Interestingly, there was no correlation between patient age and mean area of cartilage mineralization observed in this population (Figures 1C and 1D) (p = 0.342, p = 0.070). There was also no significant correlation between patient gender and mean area of cartilage mineralization, neither for the tangential cut articular cartilage nor for the slab specimens (tangential cut cartilage: female 1.30% ± 0.17 SE, male 1.16% ± 0.23 SE, p = 0.556; slab specimens: female 3.72% ± 0.38 SE, male 3.65% ± 0.56 SE, p = 0.909).

### Cartilage mineralization correlated with the degree of histologic changes

**Figure 2:**
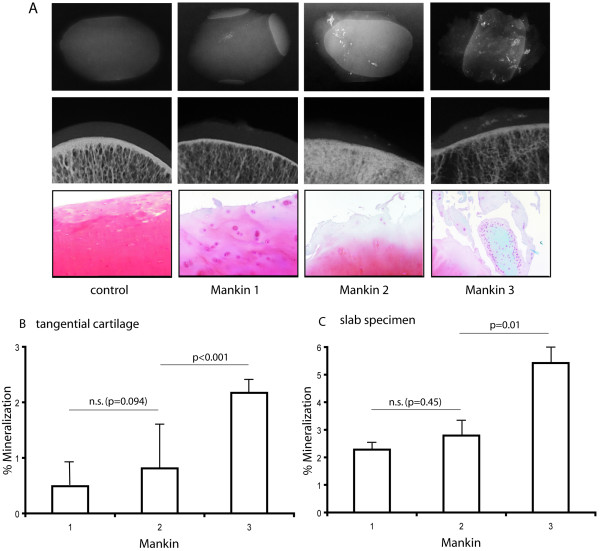
**Cartilage mineralization and correlation with the degree of histologic changes**. Histological assessment was performed on tangential cut cartilage using a modified Mankin scoring system. Three representative histological sections from each Mankin grade and one from the control group are shown with their corresponding DCRs. Whereas the 10 controls show intact articular cartilage with no mineralization detected, an increase in matrix mineralization with further cartilage destruction was observed by DCR (A). A statistically significant positive correlation between OA grade and articular cartilage mineralization for both the slab specimens and the tangential cut articular cartilage was observed in specimens graded as Mankin II and III (I-II: p = 0.036; II-III: p = 0.001) (B, C).

The patients were divided into three groups according to the Mankin classification (mild I, moderate II, severe III), as described in *Methods*. This population demonstrated a statistically significant positive correlation between Mankin OA grade and articular cartilage mineralization for both the slab specimens and the tangential cut articular cartilage. This is illustrated in Figure 2A, where four representative patients for each Mankin grade are shown with their corresponding DCRs. Quantitative analysis of all 80 samples showed a significant increase in cartilage mineralization between cartilage specimens graded as Mankin II and III. The comparison between Mankin I and II did not show a significant increase in cartilage mineralization (Figures 2B and 2C). The ten controls did not present any mineralization, neither in the slab specimens nor in the tangential cut articular cartilage. They also did not present any mineralizations or any signs of OA on the X-rays. In this context, no correlation was found between patient age and radiological OA grade according to the Kellgren classification (Kellgren II: 70.8 years at mean ± 2.5 SE; Kellgren III: 70.9 years at mean ± 1.7 SE; Kellgren IV: 69.2 years at mean ± 1.7 SE). In addition, no correlation was found between patient age and HHS or between patient age and histological OA grade according to the Mankin classification.

In a subset of 30 patients, there was high interobserver and intraobserver correlation with regard to the mineralized area of the tangential and slab cartilage specimens (kappa>0.95, p < 0.01).

### Ultrastructural analysis demonstrates primary deposition of BCPs

**Figure 3:**
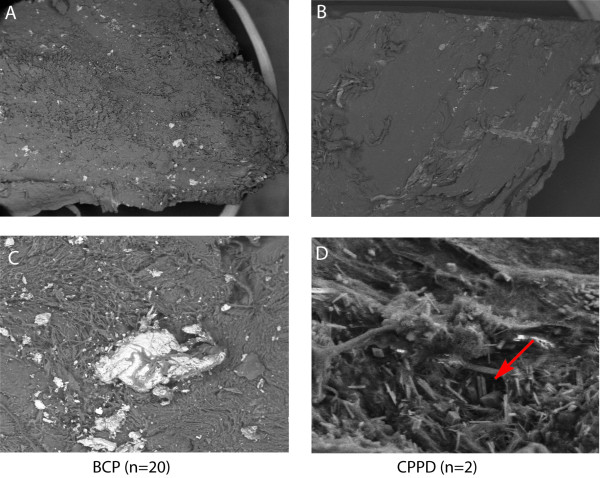
**Electron microscope analysis of cartilage mineralization**. FE-SEM analysis in combination with EDX was performed on a subset of 20 consecutive specimens out of the total 80. All areas of mineralization identified by DCR were proven to be calcium phosphate aggregates using this technique. The FE-SEM studies detected one nearly amorphous (A, C) and one idiomorphic mineral (B, D) phase with different chemical compositions. The two mineral phases contained calcium-phosphate ratios similar to BCP (A, C) and CPPD crystals (B, D). BCP crystals were found in all patients, CPPD crystals additionally in two patients. Panel A show a cartilage specimen with apatite calcification at 15× magnification; panel C the same specimen at 500× magnification. Panel B (15×) shows a patient with CPPD crystals. In panel D (500× magnification), the typical rhomboid CPPD crystals (arrow) are visible.

After cartilage specimens were analyzed by DCR, electron microscope analysis was performed on a subset of the first 20 consecutive tangential-cut cartilage specimens out of the total 80. The mineralization in all patients identified by DCR was proven to be calcium phosphate aggregates with this technique. FE-SEM studies suggest the presence of at least two phases, one nearly amorphous and one idiomorphic mineral phase, with different chemical compositions. Ten different spots on every specimen were selected for chemical measurement of the crystals. The two mineral phases contained calcium-phosphate ratios similar to apatite (1.61 ± 0.073, range 1.48-1.71) or CPPD (1.03 ± 0.068, range 0.96-1.24) crystals. Apatite-like mineral phases were detected in all patients, while CPPD crystals were present in only two patients (Figure 3). Viewing the FE-SEM pictures there were more BCP than CPPD crystals in these two patients, but an exact quantification was not possible.

These two patients with additional CPPD crystals had also primary OA of their hips with no further metabolic disorders possibly relevant for mineral deposition. Especially, there was no evidence of hypercalcemia, hyperparathyreodism, hemochromatosis or disturbances of the vitamin D or phosphate metabolism. Also no family history of crystal arthropathies was present.

## Discussion

In this prospective study, mineralization of articular cartilage was found in all patients undergoing total hip replacement; no calcification was present in the young control patients without a history of or histological evidence for OA. In addition, we found a inverse correlation between articular cartilage mineralization and clinical symptoms. Also, a strong positive correlation between cartilage mineralization and histological OA grade has been established. This is analogous to recent findings concerning articular cartilage mineralization of OA knees, where clinical and histological influence on articular cartilage mineralization was also demonstrated [10].

There was no correlation between the ages of the patients an amount of mineralization, though there was a trend toward significance for the age and the DCR of the tangential cartilage. But there is a clear result with the slab specimen of a correlation between age and mean area of mineralization definitely being excluded. The DCR of the slab specimens are more accurate because the total amount of measured cartilage is higher than in the tangential cut cartilage.

Two recent studies have concluded that age rather than OA is the predominant factor driving progressive pathologic mineralization in articular cartilage [9] and that mineralization does not necessarily worsen OA progression [16]. Those results contrast in part with our findings. Both authors concluded that cartilage mineralization may be a precursor to increased OA rather than a result of this degenerative condition.

The results of our study suggest that mineralization plays in important role in OA because it is strongly associated with histological and clinical OA severity. These results are in direct contrast to the findings from Mitsuyama and colleagues [9]. They reported that mineralization was seen to increase with age and between normal and mildly fibrillated cartilage only. No differences were observed in the mineralization level if OA progressed beyond that mild grade. No simple explanation for this paradox is apparent. Patient selection, methods for detecting cartilage mineralization, and a long list of possible artifacts, for example the use of cartilage provided by tissue banks or obtained after autopsy with the risk of postmortal artifacts, may play a role.

From this study we cannot conclude if the calcification was present before OA began or vice versa. But two facts may indicate that mineralization is a result of OA and is not merely age related: The controls without OA did not show any mineralization, either in the contact or in the FE-SEM analysis. Further, there was no correlation between patient age and the amount of matrix mineralization in this population. Though this population was carefully selected, a relationship between age and mineralization would have been expected if aging was the reason for cartilage mineralization.

As a further limitation of the study, the control group was not optimal. The primary intention of having the control group was to exclude measurements of artifacts. In 10 patients with no evidence of OA we could demonstrate that the methods used to detect mineralization were able to discriminate clearly between mineralized and nonmineralized cartilage. The control group was not intended to be an age-matched group. The correlation between age and mineralization found in this population was not controlled by the non-OA group.

An important observation is that the amount of cartilage mineralization correlates with clinical symptoms, giving reason to develop a therapeutic strategy that reduces mineralization in the cartilage to relieve symptoms of OA. As a limitation of this study, the OA material studied was not a random sample in that it was obtained from patients who presented symptoms.

The mechanism whereby BCP mineralization can cause tissue damage and inflammatory joint pain is stimulation of cytokine production in synovial fibroblasts (SF): BCP are observed to be an amplifier of PGE_2 _production through induction of the COX enzymes and the proinflammatory cytokine IL-1β in SF and thereby contribute to the severity of the synovial response [17]. But the chondrocytes themselves may also play an active role in cartilage destruction induced by microcrystals: Up-regulation of matrix metalloproteinase production by chondrocytes [18] and direct activation of chondrocytes to induce IL-1β and iNOS gene expression and NO production by BCP (OCP) crystals was observed [19].

The technique used to identify mineralization combines three different approaches and so minimizes artifacts. The challenge in obtaining a tangential-cut cartilage specimen is not touching the subchondral bone or the deep calcified cartilage area. Osteophytes can also produce artifacts. But bone artifacts can be distinguished from cartilage calcification because of the structure shown by DCR. The slab-cut specimens have a risk of showing false mineralization produced by bone dust. In the FE-SEM analysis, these artifacts can be easily excluded by the fact that cartilage mineralization is surrounded by cartilage matrix or strongly connected to the collagen fibers. Subchondral bone or mineralization from the deep cartilage zone can easily be distinguished from cartilage mineralization. In the FE-SEM analysis, more mineralizations and crystals are visible than are detected by the DCR technique. Electron microscopy of the first 20 specimens without further selection revealed that BCP, like apatite, is the prominent mineral, while CPPD crystals occur infrequently; only two patients presented CPPD crystals.

Contact radiography using DCR on cartilage specimens from a well-defined joint area is a sensitive technique for detecting mineralizations, but not for analyzing them. It gives an accurate assessment of the amount of mineralization, confirmed in this study by the use of FE-SEM [10,20,21].

## Conclusions

The fact that articular cartilage mineralization was found in all specimens from patients with OA of the hip has a significant impact on diagnostic strategies, treatment concepts, and our understanding of OA. Mineralization of articular cartilage is generally present in OA of the hips, not only as a subentity in certain progressive forms of OA. Agents that reduce crystal growth, such as phosphocitrate [22] or first-generation bisphosphonates, [23] should be considered as new therapeutic strategies to inhibit further BCP crystal formation and to reduce clinical symptoms in OA.

## Competing interests

The authors declare that they have no competing interests.

## Authors' contributions

This work was supported by "Deutsche Arthrosehilfe e.V", Neue-Welt-Str. 4-6, 66740 Saarlouis, Germany, grant nr: p77-a117-Rüther-EP2-fuer1-knie-ko--49k-2006. The founders had no role in study design, data collection and analysis, decision to publish or preparation of the manuscript.

MF carried out most of the experiments, coordinated, designed and wrote the study, ON carried out the Harris Hip score and coordinated the sample collection, LL carried out the FE-SEM analysis, FS carried out the DCR, CL coordinated and desiged th study and WR participated in its design and coordination and helped to draft the manuscript. All authors read and approved the final manuscript.

## Pre-publication history

The pre-publication history for this paper can be accessed here:

http://www.biomedcentral.com/1471-2474/10/166/prepub
